# Supplementary material for: Complexome profiling of the Chlamydomonas psb28 mutant reveals TEF5 as an early PSII assembly factor
Source: Plant Cell. 2025 Mar 17;37(6):koaf055. doi: 10.1093/plcell/koaf055 (PMC12164590; doi:10.1093/plcell/koaf055)
Supplement: koaf055_Supplementary_Data [file koaf055_supplementary_data.zip › psb28_interactive.zip!/plots/intensity/qualityPlots/peptides/EFP1_Cre16.g691000.t1.2.html]

### Cre16.g691000.t1.2

Trivial name: EFP1  
SeqCoverage [median]: 15.30  
Mass: 23.88 kDa  
  
 

| Sequence | MissedCleavage | Mass [Da] | wtIntensity | muIntensity | Score |
| --- | --- | --- | --- | --- | --- |
| AGEQVNTADVQKR | 1 | 1414.72 | 585.44 | 692.80 | 82.85 |
| TGQYLSR | 0 | 823.42 | 0.00 | 602.19 | 75.21 |
|  |  |  | 585.4 | 1295.0 |  |
